# Chemical composition of *Luffa aegyptiaca* Mill., *Agave durangensis* Gentry and *Pennisetum* sp.

**DOI:** 10.7717/peerj.10626

**Published:** 2021-01-22

**Authors:** Oswaldo Moreno-Anguiano, Artemio Carrillo-Parra, José G. Rutiaga-Quiñones, Christian Wehenkel, Marín Pompa-García, Francisco Márquez-Montesino, Luis F. Pintor-Ibarra

**Affiliations:** 1 Programa Institucional de Doctorado en Ciencias Agropecuarias y Forestales, Universidad Juárez del Estado de Durango, Durango, Durango, Mexico; 2 Instituto de Silvicultura e Industria de la Madera, Universidad Juárez del Estado de Durango, Durango, Durango, Mexico; 3 Facultad de Ingeniería en Tecnología de la Madera, Universidad Michoacana de San Nicolás de Hidalgo, Morelia, Michoacán, Mexico; 4 Facultad de Ciencias Forestales, Universidad Juárez del Estado de Durango, Durango, Durango, Mexico; 5 Departamento de Química, Universidad de Pinar del Río, Pinar del Río, Cuba

**Keywords:** *Agave durangensis* bagasse, *Luffa aegyptiaca*, *Pennisetum* sp., Lignin, Holocellulose, Extractives, Ash

## Abstract

The particleboard industry faces problems of wood shortage, which has led to the use of non-wood lignocellulosic materials. Furthermore, there is also interest in looking for materials that improve their physical and mechanical properties. The species *Luffa aegyptiaca* Mill. (fruit), *Agave durangensis* Gentry (bagasse) and *Pennisetum* sp. (plant, leaves and stem) could be used in the elaboration of wood-based particleboards. The aim of this study is to determine the feasibility of using these materials to produce particleboards in accordance with their chemical composition. Five materials were studied, *A. durangensis* (bagasse), *L. aegyptiaca* (fruit) and *Pennisetum* sp. (whole plant, leaves and stem). Extractives, holocellulose, Runkel lignin and ash content was determined. The pH of the fibers was also measured and a microanalysis of the ash was performed. ANOVA and Kruskal-Wallis tests were carried out, in addition Tukey and Dunn tests for group comparison were performed. *Pennisetum* sp. leaves presented the highest total extractives and ash content, while *L. aegyptiaca* fruit and *A. durangensis* bagasse had the highest both content of holocellulose and Runkel lignin respectively. The lowest pH was presented by the *L. aegyptiaca* fruit, while the highest was from the *Pennisetum* sp. stem. The element with the greatest presence in the five materials was potassium, except in *A. durangensis* bagasse showing calcium. *L. aegyptiaca* fruit has better characteristics to be used in particleboards with greater mechanical resistance because of its higher holocellulose content. However, *Pennisetum* sp. (plant, leaves and stem) could be used to make particleboards with high resistance to water absorption.

## Introduction

Particleboards as composite materials are wood-based engineering products with various applications. They are manufactured with wood particles and synthetic resins or other binders (Baharoglu et al., 2013). However, the particleboard industry is looking for new renewable lignocellulosic materials that replace or complement wood and also improve the physical and mechanical properties of the boards (Scatolino et al., 2017). The properties of the individual components and the compatibility between them influence the mechanical properties of composite materials (Sheshmani, Ashori & Farhani, 2011). The chemical composition of the particles has an important role in the physical and mechanical performance of the particleboards (Klímek et al., 2016). The complex relationship between the chemical composition and particleboard properties is not yet fully understood (Klímek et al., 2016). However, some relationships between some properties of the chemical composition of the lignocellulosic material and the characteristics of the particleboard are known. Extractives cannot absorb water and they are comprised of tannins, pectins, fats, waxes, gums, essential oils and volatile materials (Gwon et al., 2010). The extractives improve the water resistance of the boards (Nemli & Aydin, 2007). The effect of the chemical composition on the gluing quality is mainly related to the extractives concentration on the wood surface and the ash content (Faria et al., 2019). However, extractives make difficult the bonding and decrease the internal bond between the particles (Nemli et al., 2004), thus the mechanical properties decrease (Ayrilmis et al., 2009).

The level of pH has an important role in developing good bonding between resin and particle which results in enhanced panel properties. pH values of lignocellulosic fibers and resin are critical to have a good bonding between particles, which influences both the physical and mechanical properties of the panels (Kalaycioglu, Deniz & Hiziroglu, 2005).

The low holocellulose content of lignocellulosic material can restrict adhesion between particles, and therefore reduce their mechanical properties. However, the reduction in holocellulose content may result in less water absorption (Klímek et al., 2016). The holocellulose can absorb water since it contains hydroxyl groups and a lower lignin content leads to higher water absorption (Nourbakhsh, Baghlani & Ashori, 2011). Intensive degradation of celulloses and lignin decrease mechanical quality of the boards (Widyorini et al., 2005).

Silicon may influence the bonding (Hashim et al., 2011). The presence of Ca and Cl could improve the thickness swelling and water absorption of the panels (Ferrández-García et al., 2017).

Studies of various non-wood lignocellulosic materials have been carried out to determine their viability in the elaboration of particleboards, such as sorghum stalks (Khazaeian, Ashorib & Dizaj, 2015), peach peel residues (Sahin et al., 2017), cane bagasse (Jonoobi et al., 2016) and stems of *Miscanthus* (Klímek et al., 2018). Despite the research realized, there are still a large number of non-wood lignocellulosic materials that have not been studied and could be used in the elaboration of particleboards. *Pennisetum* sp. (maralfalfa), the fruit of the *Luffa aegyptiaca* Mill. (sponge gourd) and the bagasse of *Agave durangensis* Gentry are some of the materials that could be utilized for manufacturing particleboards.

The chemical composition has been important in the research of lignocellulosic materials. In the genus Agave, the chemical composition has been determined in some species, for example, in *A. americana* leaf fibers, where the possibility of use these fibers in the textile industry was explored (Oudiani et al., 2009). The chemical composition of *A. tequilana* leaf fibers has also been studied for its potential use as a raw material for biofuels (Yang et al., 2015; Avila-Gaxiola et al., 2016). The chemical composition of the Luffa genus has been determined to evaluate the possible use of Luffa in the manufacturing of biocomposites (Tanobe et al., 2005) and to obtain cellulose nanocrystals (Siqueira, Bras & Dufresne, 2010). In the genus Pennisetum, chemical composition has been determined in works related to biofuels (Del Río et al., 2012), fiber and paper (Daud et al., 2014) and cellulose nanocrystals (Lu et al., 2014).

The *Pennisetum* sp. is a perennial grass that can reach a height of 4 m and its stem a diameter of 2–3 cm. It has a yield of up 210 tons per hectare (Orihuela-Porcayo & Cuevas-Ocampo, 2014). It has been used as animal feed, however, it has lower nutritional quality than others animal feed (Guerra-Medina et al., 2015). Agave bagasse is a waste that is generated in large quantities in the Mezcal industry and represents a pollution problem due to its handling and as a fire source. In the state of Durango, Mexico, there are 29 species of genus Agave (González-Elizondo et al., 2009). *Agave durangensis* is one of the most important because of its success in the Mezcal industry (Almaraz-Abarca et al., 2013). The production of one liter of mezcal generates 15–20 kg of bagasse (Ilangovan et al., 2000). In the state of Durango, Mexico, 178,625 l of mezcal were produced in 2019 (CRM, 2020), thus, it was estimated that in this year there was a production of 2,679 to 3,572 tons of wet base agave bagasse. *L. aegyptiaca* is a tropical plant of the Cucurbitacea family. It has a fruit with a fibrous vascular system (Boynard & D’Almeida, 2000). Aurin & Rasco (1988) reported a yield of luffa fruits of 37.1 ton/ha.

Therefore, the study aim is to determine the chemical composition and extractives of *A. durangensis* bagasse, *L. aegyptiaca* fruit, *Pennisetum* sp. leaves, stem and whole plant in order to determine the feasibility to produce particleboards. We expected good chemical characteristics of these five materials for particleboards.

## Materials and Methods

### Origin and preparation of raw material

Grass samples *Pennisetum* sp. were obtained from the experimental field Valle del Guadiana of the National Institute of Forestry, Agricultural and Livestock Research (INIFAP, Campus Durango, Mexico). From this material, three different materials were obtained, the leaves, the stem and the whole plant. The whole plant had a ratio of 70% stem and 30% leaves based on weight. *A. durangensis* bagasse was donated by a Mezcal company of the Durango region, in the state of Durango, Mexico. The bagasse used in this work was fermented for 10 days and distilled for 20–30 h. It was not washed and it was used as it came from the industry. *L. aegyptiaca* fruit was recollected in the municipality of Tacámbaro, Michoacán, Mexico.

The material was dried in the shade at room temperature for four weeks and milled in a Wiley mill type. The flour obtained was sieved using 40 (0.425 mm) and 60 (0.250 mm) mesh, using the material retained in the 60 mesh for analysis. The material was stored in airtight containers for further analysis.

### Extractives content

The content of extractives was determined according to Mejía-Díaz & Rutiaga-Quiñones (2008). Successive extraction was carried out in Soxhlet equipment with the following solvents cyclohexane, acetone, methanol and finally hot water under reflux, for periods of 6 h. The number of extractions depended on the material and the solvent (Table 1) and it was performed until the constant weight of the flask, that is to say, when the flask weight did not increase anymore due to the absence of extracts. The solvents were recovered using a rotary evaporator and the content of extractives was determined by gravimetry. The flour resulting from the extraction was called extractives free material and was used to determine lignin and holocellulose.

**Table 1:** Number of extractions per solvent for each material.

Material	Extracts			
	Cyclohexane	Acetone	Methanol	Hot water
*A. durangensis* bagasse	2	2	3	4
*L. aegyptiaca* fruit	1	1	2	1
*Pennisetum* sp. plant	3	2	5	2
*Pennisetum* sp. leaves	7	1	5	2
*Pennisetum* sp. stem	1	1	6	2

### pH determination

The methodology described by Sandermann & Rothkamm (1959) was used to determine the pH of the fibers. A total of 2 g of flour were used without extracting from each material, which were placed in 20 ml of distilled water at room temperature. With a potentiometer five readings were taken, at the beginning, at 5 min, at 4, 24 and 48 h, considering the last reading for the final analysis. The first four readings were taken only as a reference.

### Holocellulose, Runkel lignin and ash content

The holocellulose content was determined by the method of Wise, Murphy & D’Addieco (1946). One gram of each extractives free material was placed in a flask. Sodium chlorite (NaClO_2_) (0.3 g) and glacial acetic acid (CH_3_COOH) (0.2 ml) were added. The mixture was taken in a water bath at 75 °C during 4 h, adding the same amount of NaClO_2_ and CH_3_COOH every hour. It was washed and filtered with a liter of cold distilled water and 20 ml of acetone. Then, it was dried at 40 °C to constant weight and the holocellulose content calculating by gravimetry as percentage of dry weight of extractive free material.

The method of Runkel & Wilke (1951) was used to determine the lignin content. A total of 50 ml of 72% sulfuric acid (H_2_SO_4_) and 50 ml of 40% hydrobromic acid (HBr) were added to 2 g of each extractive free material. It was left to rest for 2 h. Then, it was washed to remove the acid with hot distilled water and filtered. The lignin filtered was dried at 100 °C in an oven to a constant weight. Then, the lignin was determined gravimetrically as a percentage of dry weight of extractive free material.

The ash (inorganic substances) was calculated according to the standard T 211 om-93 (TAPPI, 2000). Two g of flour were placed in a nickel crucible and brought to a muffle at 525 °C for 2 hours. The crucible was cooled in a desiccator. The ash content was calculated by gravimetry as a percentage of total dry weight.

### Ash microanalysis

Microanalysis of the ash obtained was carried out in an X-ray energy dispersion spectrometer (EDS), coupled to a Scanning Electron Microscope (SEM) brand Jeol model JSM6400. The operating conditions for the analyzes were 20 kV and 8.5 s (Téllez-Sánchez et al., 2010).

The studies were done in triplicate in each analysis, except for the holocellulose that was determined in four replicates.

### Statistical analysis

Data of each variable were analyzed using a completely randomized design. The type of material was the factor. The data were analyzed with the R Studio software (R Core Team, 2013). The normality was checked with the Shapiro-Wilk test. An analysis of variance (ANOVA) (significant level = 0.05) was carried out in data with normal distribution and the comparison of means by the Tukey test was developed when factors were statistically different. In data without normal distribution, a Kruskal-Wallis analysis (significant level = 0.05) was performed and the groups were compared by Dunn’s test with Bonferroni correction.

## Results

### Extractives Content

**Figure 1:**
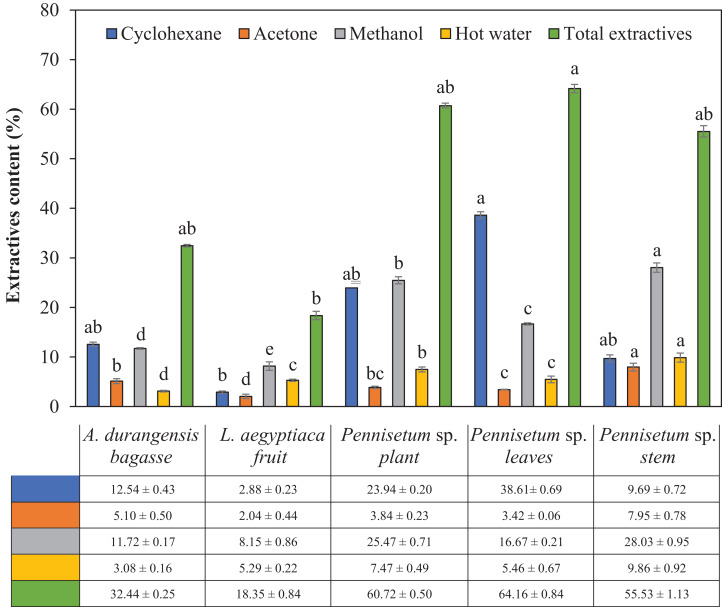
Extractives content (%) of *L. aegyptiaca* (fruit), *A. durangensis* (bagasse) and *Pennisetum* sp. (plant, leaves and stem). Different lowercase letters indicate statistically significant difference among materials by Tukey test (*p* < 0.05). The values under the graph represent the means and standard deviations.

There were significant differences in the content of total extractives and by solvent among the materials analyzed (Table 2). Fruit of *L. aegyptiaca* was the material with the lowest extractive content, while the *Pennisetum* sp. leaves recorded the highest content. Using cyclohexane, there was only a significant difference between *L. aegyptiaca* fruit and *Pennisetum* sp. leaves. Using acetone, *L. aegyptiaca* fruit and *Pennisetum* sp. stem only showed significant differences among themselves and with the rest of the materials. Using methanol, there were significant differences between the five materials. Using hot water only, here was only a significant difference in the content of total extractives in *L. aegyptiaca* fruit and the *Pennisetum* sp. leaves. The highest content of total and cyclohexane extractives was presented by *Pennisetum* sp. leaves, while the *Pennisetum* sp. stem did it for the other three solvents (Fig. 1).

**Table 2:** Shapiro–Wilk, Kruskal–Wallis and ANOVA (*p* < 0.05) tests of the extractives content by solvent and total of *L. aegyptiaca* (fruit), *A. durangensis* (bagasse) and *Pennisetum* sp. (plant, leaves and stem).

Extractives	Shapiro–Wilk test		Kruskal–Wallis test		ANOVA test	
	Statistic	*p-*Value	Chi-squared	*p-*Value	*F* value	*p*-Value
Cyclohexane	0.871	0.034	*13.500*	*0.009*		
Acetone	0.914	0.157			*67.890*	*3.29 × 10* ^*−7*^
Methanol	0.883	0.053			*500.370*	*1.8 × 10* ^*−11*^
Hot water	0.948	0.500			*60.760*	*5.59 × 10*^*−7*^
Total extractives	0.817	0.006	*13.500*	*0.009*		

**Note:**

Italic values indicate statistical differences (*p* < 0.05) among materials.

### pH of fibers

**Figure 2:**
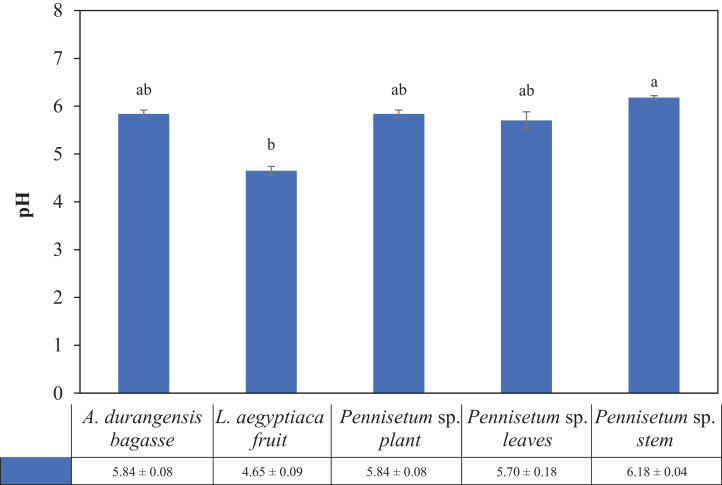
pH of *L. aegyptiaca* (fruit), *A. durangensis* (bagasse) and *Pennisetum* sp. (plant, leaves and stem). Different lowercase letters indicate statistically significant difference among materials by Tukey test (*p* < 0.05). The values under the graph represent the means and standard deviations.

There were significant differences in pH between *Pennisetum* sp. stem (6.18) and *L. aegyptiaca* fruit (4.65), but they did not present differences with *A. durangensis* bagasse (5.84), *Pennisetum* sp. plant (5.84) and *Pennisetum* sp. leaves (5.70) (Table 3; Fig. 2).

**Table 3:** Shapiro–Wilk and Kruskal–Wallis (*p* < 0.05) tests of pH of *L. aegyptiaca* (fruit), *A. durangensis* (bagasse) and *Pennisetum* sp. (plant, leaves and stem).

	Shapiro–Wilk test		Kruskal–Wallis test	
	Statistic	*p-*Value	Chi-squared	*p-*Value
pH	0.799	0.003	*11.433*	*0.022*

**Note:**

Italic values indicate statistical differences (*p* < 0.05) among materials.

### Holocellulose, Runkel lignin and ash content

**Figure 3:**
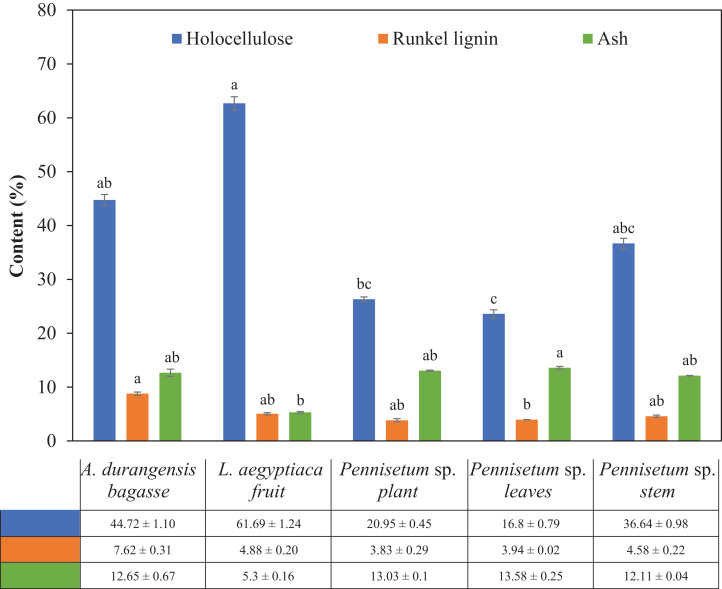
Holocellulose, Runkel lignin and ash content (%) of *L. aegyptiaca* (fruit), *A. durangensis* (bagasse) and *Pennisetum* sp. (plant, leaves and stem). Different lowercase letters indicate statistically significant difference among materials by Tukey test (*p* < 0.05). The values under the graph represent the means and standard deviations.

The fiber of the *L. aegyptiaca* fruit had the highest holocellulose content and *Pennisetun* sp. leaves had the smallest. The five materials showed significant differences in holocellulose content. *A. durangensis* bagasse and *Pennisetum* sp. leaves had significant differences in the content of Runkel lignin, but not with the other three materials. *A. durangensis* bagasse had the highest Runkel lignin content. The lowest ash content was presented by *L. aegyptiaca* fruit, which had a significant difference with *Pennisetum* sp. leaves (Table 4; Fig. 3).

**Table 4:** Shapiro–Wilk, Kruskal–Wallis and ANOVA (*p* < 0.05) tests of holocellulose, Runkel lignin and ash of *L. aegyptiaca* (fruit), *A. durangensis* (bagasse) and *Pennisetum* sp. (plant, leaves and stem).

	Shapiro–Wilk test		Kruskal–Wallis test	
	Statistic	*p-*Value	Chi-squared	*p-*Value
Holocellulose	0.884	0.020	*18.286*	*0.001*
Runkel lignin	0.777	0.002	*12.567*	*0.014*
Ash	0.663	1 × 10^−3^	*12.000*	*0.017*

**Note:**

Italic values indicate statistical differences (*p* < 0.05) among materials.

### Microanalysis of ash

**Figure 4:**
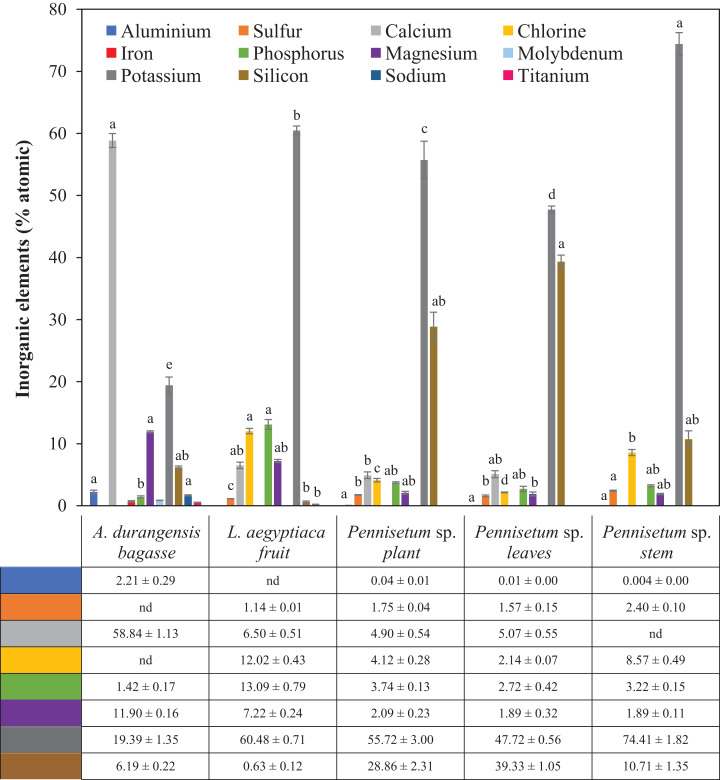
Microanalyses of ash (% atomic) of *L. aegyptiaca* (fruit), *A. durangensis* (bagasse) and *Pennisetum* sp. (plant, leaves and stem). Different lowercase letters indicate statistically significant difference among materials by Tukey test (*p* < 0.05). The values under the graph represent the means and standard deviations. Iron (0.70 ± 0.12), molybdenum (0.85 ± 0.02) and titanium (0.53 ± 0.03) were found only in *A. durangensis* bagasse. Sodium was found in *A. durangensis* bagasse (1.66 ± 0.09) and *L. aegyptiaca* fruit (0.20 ± 0.03) nd, not determined.

*Pennisetum* sp. stem and *A. durangensis* bagasse had significant differences in the atomic % of potassium. There was significant difference in atomic % of silicon between *Pennisetum* sp. leaves and *L. aegyptiaca*, which had the lowest presence of silicon. Iron, molybdenum and titanium were only found in *A. durangensis* bagasse. The element with the largest presence in *A. durangensis* bagasse was calcium, while potassium was for others (Table 5; Fig. 4).

**Table 5:** Shapiro–Wilk, Kruskal–Wallis and ANOVA (*p* < 0.05) tests of ash microanalyses of *L. aegyptiaca* (fruit), *A. durangensis* (bagasse) and *Pennisetum* sp. (plant, leaves and stem).

	Shapiro–Wilk test		Kruskal–Wallis test		ANOVA test	
	Statistic	*p-*Value	Chi-squared	*p-*Value	*F* value	*p*-Value
Aluminium	0.601	1.1 × 10^−4^	7.666	0.053		
Sulfur	0.895	0.137			*96.610*	*1.27 × 10*^*−6*^
Calcium	0.586	8.25 × 10^−5^	*9.462*	*0.024*		
Chlorine	0.876	0.078			*457.400*	*2.77 × 10*^*−9*^
Phosphorus	0.684	1.7 × 10^−4^	*13.233*	*0.010*		
Magnesium	0.744	7.7 × 10^−4^	*11.733*	*0.019*		
Potassium	0.882	0.051			*417.800*	*4.45 × 10*^*−11*^
Silicon	0.854	0.020	*13.500*	*0.009*		
Sodium	0.730	0.013	*3.857*	*0.049*		

**Note:**

Italic values indicate statistical differences (*p* < 0.05) among materials.

## Discussion

### Extractives content

The total extractives content of *A. durangensis* bagasse and *L. aegyptica* fruit were similar to those reported by other authors who used the same extraction sequences for non-wood lignocellulosic materials. Pintor-Ibarra et al. (2018) obtained values from 21.82 to 35.69% of total extractives in root, leaves and stem of *Eichhornia crassipes*. While the plant, leaves and stem of *Peninisetum* sp. presented contents closer to *Agave durangensis* leaves (51.69%) (Contreras-Hernández et al., 2018). However, using different methods to this work, the total extractives content of the five materials studied is larger than reported both by Scatolino et al. (2017) with 26.24% and Hidalgo-Reyes et al. (2015) with 5.3% for coffee parchment and bagasse from *A. angustiflora*, respectively. The differences between the contents of extractives in lignocellulosic materials are due to differences in the species and the geographical location of the plant (Hardell & Nilvebrant, 1999).

The high content of extractives in the materials for the manufacture of particleboards has advantages and disadvantages. This is due to the fact that extractives have a significant influence on the properties of lignocellulosic materials, according to the quantity and type of extractives present in the materials (Shebani, Van Reenen & Meincken, 2008). A disadvantage for high extractives content it is the reduction of adhesion of the interface and, therefore, reduced resistance of internal adhesion (Sheshmani, Ashori & Farhani, 2011).

Regularly extractives alter the properties of the lignocellulosic material, which in turn modifies its adhesion properties (Gadhave, Mahanwar & Gadekar, 2017; Hse & Kuo, 1988). Four of the five materials studied, had low content of non-polar extractives, with the exception of the leaves of *Pennisetum* sp. Materials with a higher content of non-polar extractives could present problems in the gluing quality. This is due to the fact that the adhesion is reduced by the migration of the extracts to the surface after high temperatures (Hancock, 1963; Faria et al., 2019).

Waxes and phenolic resin are incompatible, since they have different polarities, which means that the adhesive reduces its ability to wet and penetrate the lignocellulosic material (Hse & Kuo, 1988). Because of this, when phenolic resins are used, it is convenient to use lignocellulosic materials with low content of non-polar extractives (waxes) to increase the gluing quality (Thomas, 1959). When urea formaldehyde is used as an adhesive, nonpolar extractives also reduce the wettability of the material (Jordan & Wellons, 1977) resulting in minor resistance to internal bond of the boards (Hse & Kuo, 1988).

Instead, an advantage is that a kind of extractives are hydrophobic substances and therefore can have a role similar to waxes in the manufacture of particleboards increasing their resistance to water absorption (Sheshmani, Ashori & Farhani, 2011). Silva et al. (2018) observed that by increasing the proportion of castor husk particles (which had high content of extractives) in pine-based panels, the resistance to water absorption increased.

A lignocellulosic material that was extracted absorbs more water and swells more than a material that was not extracted, due to the increase in the disposition of the spaces in which there were extractors and the increase in the diffusion coefficient (Nzokou & Kamdem, 2004).

In lignocellulosic materials, hydroxyl groups are responsible for the interaction with water molecules through hydrogen bonds. The availability of free hydroxyl groups on the surface of the lignocellulosic material particles affects the water absorption of wood-based composite materials (Sheshmani, Ashori & Farhani, 2011). When lignocellulosic material is not extracted, these hydroxyl groups are blocked and therefore water absorption is lower (Kim, Harper & Taylor, 2009). Extractives can block cells and reduce contact between hydroxyl groups of materials due to their diffusion on the surface of the cellulosic material (Bledzki et al., 2005).

The penetration of water by the capillarity action to the deepest parts of lignocellulosic materials is reduced because the lumens of the cells are occupied by the extractives, thus, water absorption occurs only on the surface (Sheshmani, Ashori & Farhani, 2011), therefore, the absorption of water by the particleboards decreases.

According to Nourbakhsh, Baghlani & Ashori (2011), the admission of water is proportional to the amount of hydroxyl groups. The absorption of water and the swelling in thickness of the particleboards proportionally differ with the increase of extractives content (Nasser, 2012).

### Holocellulose, Runkel lignin and ash

The holocellulose content of *A. durangensis* bagasse was lower than reported by Hidalgo-Reyes et al. (2015) in bagasse of *A. angustiflora* (82.12%), However, it was higher than *A. durangensis* leaves (20.3%) (Contreras-Hernández et al., 2018). The holocellulose content of *A. durangensis* bagasse was similar to *A. sisalana* bagasse (48.2%) (Lima et al., 2013) and leaves of *A. atrovirens* Karw (43.5%) (Hernández-Hernández et al., 2016). *L. aegyptica* fruit had a lower holocellulose content than *L. cylindrica* (84.84%) (Akgül et al., 2013). Nevertheless, it had a higher holocellulose content than both rice straw (57.64%) and pseudostem of the banana plant (50.92%) obtained by Wang et al. (2018) and Guimarães et al. (2009), respectively. The holocellulose content of *Pennisetum* sp., both leaf, stem and whole plant was lower than *P. purpureum* (60.51%) (Santos, Silva & Queiroz Filho, 2001).

Cellulose and hemicellulose contain a large number of hydroxyl groups (Pirayesh & Khazaeian, 2012), which are responsible for the bond between particles and polar adhesive polymers (Ayrilmis et al., 2009). Thus, the low holocellulose content of the materials could reduce the bonding between the particles, affecting the mechanical properties. However, a low holocellulose content and thus a low presence of hydroxyl groups, results in a lower water absorption (Klímek et al., 2016).

The lignin content of the five materials was lower than of *A. angustiflora* bagasse (17.9%) (Hidalgo-Reyes et al., 2015), rice straw (14.7%) (*Wang et al., 2018)*, pseudostem of the banana plant (17.44%) (*Guimaraes et al., 2009*), coffee parchment (28.3%) (Scatolino et al., 2017), and *A. durangensis* leaves (14.15%) (Contreras-Hernández et al., 2018).

Lignin provides the mechanical strength of lignocellulosic materials (Neutelings, 2011). It is also totally amorphous and hydrophobic, so water absorption does not occur in lignin (Nourbakhsh, Baghlani & Ashori, 2011). Due to its hydrophobic nature, lignin makes plant cells impervious to water (Achyuthan et al., 2010).

The ash content of *A. durangensis* was higher than bagasse of *A. tequilana* (7.4%) (Liñán-Montes et al., 2014) and fiber of the leaves of *A. tequilana* (6.0%) (Yang et al., 2015). Tanobe et al. (2005) and Siqueira, Bras & Dufresne (2010) reported for *L. cylindrica* an ash content of 0.4% and 0.7%, respectively, which is lower than fruit of *L. aegyptiaca*. The ash content of *Pennisetum* sp., both leaf, stem and whole plant, was higher than the ash content of *P. sinese* (3.3%) (Lu et al., 2014), however, it was lower than *P. purpureum* (14.6%) (Daud et al., 2014).

The chemical composition of non-wood lignocellulosic materials depends on several factors, despite being the same species. For example, in annual plants the chemical composition varies according to the harvest time (Gunnarsson et al., 2014). Other factors that affect the differences in chemical composition are the sample preparation and the analytical methods used (Klímek et al., 2016).

### pH of fibers

The pH of *A. durangensis* bagasse, plant and leaves of *Pennisetum* sp. is similar to *Ailanthus altissima* (5.9) (Bardak, Nemli & Tyriaki, 2017) and *Populus canadensis moench* (5.97) (Baharoglu et al., 2013). *L. aegyptiaca* fruit presented a pH close to *Pinus brutia Ten*. (4.79) obtained by Baharoglu et al. (2013), but was higher than the needle litter of *Pinus sylvestris* L. (3.01) reported by Nemli, Yıldız & Gezer (2008). The pH of *Pennisetum* sp. stem was higher than the previous materials.

Nemli, Yıldız & Gezer (2008) stated that a lower pH in lignocellulosic materials may be attributed to presence of more amounts of extractives and resin acids. The acidic reaction of most tree species is caused by free acids and acidic groups easy to be separated, that is, especially by acetic acid and acetyl groups (Geffert, Geffertova & Dudiak, 2019). Variations of pH along the stem have also been associated with the presence of extractives (Campbell, Kim & Koch, 1990).

Materials with a low pH result in boards with lower resistance properties (Baharoglu et al., 2013), since the adhesive curing occurs before hot pressing when the pH value of the material is low (Nemli, Yıldız & Gezer, 2008). The acidity of the lignocellulosic material influences the cure of urea formaldehyde adhesive. When this happens the adhesive bond breaks down and decreases the adhesion and internal bonding between the particles, since the binder is cured before the particles have been compressed and, therefore, when the press closes the resin bonds obtained are broken, resulting in the layers of boards being weak and flaky (Baharoglu et al., 2013).

Urea formaldehyde (UF) is an adhesive that cures better in an acid environment (Poblete & Burgos, 2010), therefore, the five analyzed materials have good conditions to be glued with this adhesive.

### Ash microanalysis

Potassium was the element with the greatest presence, result similar to Camarena-Tello et al. (2015) for guava branches and leaves, and to Lara-Serrano et al. (2016) for root, stem and leaves of *Eichhornia crassipes*.

## Conclusions

According to their chemical composition, some of the materials studied present good conditions for manufacturing particleboards.

The *Luffa aegyptiaca* fruit, the *Agave durangensis* bagasse and *Pennisetum* sp. (plant, leaves and stem) could be used as reinforcement in the elaboration of wood particleboard according to their chemical properties. *A. durangensis* bagasse and *L. aegyptiaca* fruit could be used to produce particleboards (mixed with particles of wood or not combined with wood particles) from the point of view of their values of pH, holocellulose and lignin content. The plant, leaves and stem of *Pennisetum* sp. could be used to reinforce wood particleboards that are intended to be used in humid environments and that require less mechanical resistance because of the high content of extractives, but low content of holocellulose and lignin. On the other hand, the excessive use of *Pennisetum* sp. stem, could cause damage to the cutting tools due to its high silicon content. Nevertheless, there is needed more research to indicate the influence of inorganic elements in the manufacturing process and in the properties of particleboards.

## Supplemental Information

10.7717/peerj.10626/supp-1 Supplemental Information 1 Raw Data of the determination of chemical composition of *L. aegyptiaca* (fruit), *A. durangensis* (bagasse) and *Pennisetum* sp. (plant, leaves and stem).

## Additional Information and Declarations

### Data Availability

The following information was supplied regarding data availability: The raw data of the extractives, ash, lignin and holocellulose content of Luffa aegyptiaca (fruit), Agave durangensi (bagasse) and *Pennisetum* sp. (plant, leaves and stem), as well as microanalysis of the ash and the pH of the fibers are available as a Supplemental File.
